# Joint TITE-CRM: A Design for Dose Finding Studies for Therapies with Late-Onset Safety and Activity Outcomes

**DOI:** 10.1080/19466315.2024.2333388

**Published:** 2024-03-20

**Authors:** Helen Barnett, Oliver Boix, Dimitris Kontos, Thomas Jaki

**Affiliations:** a MRC Biostatistics Unit, University of Cambridge, Cambridge, UK; b Lancaster University, Lancaster, UK; c Bayer AG, Leverkusen, Germany; d ClinBAY, Limassol, Cyprus; e University of Regensburg, Regensburg, Germany

**Keywords:** Dose-finding, Late-onset activity, Late-onset toxicities, Model-Based, Phase I trials

## Abstract

In Phase I/II dose-finding trials, the objective is to find the Optimal Biological Dose (OBD), a dose that is both safe and shows sufficient activity that maximizes some optimality criterion based on safety and activity. In cancer, treatment is typically given over several cycles, complicating the identification of the OBD as both toxicity and activity outcomes may occur at any point throughout the follow up of multiple cycles. In this work we present and assess the Joint TITE-CRM, a model-based design for late onset toxicities and activity based on the well-known TITE-CRM. It is found to be superior to the currently available alternative designs that account for late onset bivariate outcomes, as well as being both intuitive and computationally feasible.

## 1 Introduction

In traditional drug development, safety and therapeutic activity of potential new drugs have been evaluated separately. A phase I trial first finds the maximum tolerated dose, the dose associated with some predetermined probability of observing a Dose-Limiting Toxicity (DLT). This dose is then carried forward to phase II, where therapeutic activity is evaluated, with limited borrowing of information between the evaluation of safety and activity. An alternative option is a seamless Phase I/II trial where safety and activity are evaluated simultaneously, with the aim to find the Optimum Biological Dose (OBD). The main advantage of collecting information on both outcomes is the increased chance of finding a dose that is both safe and has potential to be efficacious, by allowing for more sharing of information.

There are multiple methods that have been proposed to design such a trial with a binary safety and binary activity endpoint that range in complexity. For example the relatively simple model-assisted toxicity and efficacy interval design (STEIN) of Lin and Yin (2017), or more complex model-based designs that can jointly model the dose–toxicity and dose–activity relationship such as the utility contour of Thall and Cook (2004), or the approach based on toxicity and activity odds ratios (bCRM) by Yin, Li, and Ji (2006), or the bivariate Continual Reassessment Method for competing outcomes of Braun (2002) amongst others. These methods differ in both their approaches to inference of the bivariate (or trinary) outcomes, and the decision criteria based on this inference. In addition, approaches that model safety as binary and use a continuous marker of activity have been proposed (e.g., Nebiyou Bekele and Shen 2005; Yeung et al. 2015, 2017). Common to those proposals is the assumption that as each cohort of patients enters the trial, responses from all previous cohorts are available to inform the decision of the next dose assignment. The next cohort is assigned the best dose in order to collect more information at the current estimate of the OBD.

In oncology, where there are multiple cycles of treatment, both safety and activity outcomes may have a delayed onset. There are a limited number of phase I designs that account for late onset toxicities, but an even more limited number of designs that account for late onset outcomes in both safety and activity. The main contributions to model-based dose-finding trial designs incorporating late onset toxicities include the Time-to-Event version of the Continual Reassessment Method (TITE-CRM) by Cheung and Chappell (2000), the interval censored approach (ISCDP) of Sinclair and Whitehead (2014), and the approach of including a cycle effect in a proportional odds mixed effects model (POMM) by Doussau, Thiébaut, and Paoletti (2013). A comprehensive review of these was undertaken by Barnett et al. (2022). However, these do not take into account the therapeutic activity, the inclusion of which is not a straightforward matter. When including late-onset activity as well, there are therefore a very limited number of approaches. For example, Koopmeiners and Modiano (2014) consider binary late-onset toxicity outcomes with activity measured as survival beyond the observation window, and Altzerinakou and Paoletti (2020) consider a binary late-onset toxicity outcome with a continuous longitudinal activity endpoint. In many trial settings, both toxicity and activity are binary late-onset outcomes, with both being observable in any of the multiple cycles of treatment. For this setting, the method of jointly modeling the time-to-event of activity and toxicity (*A_T_*/*A_E_*) using survival models of Yuan and Yin (2009), the model assisted approach of Liu and Johnson (2016), and the TITE-B design of Yan et al. (2019) are available designs. However, the model-assisted design lacks the flexibility of the model based designs and neither of the two model-based designs, the *A_T_*/*A_E_* or the TITE-B, target specific toxicity or activity levels.

In this article we introduce a joint time-to-event CRM (Joint TITE-CRM), a model-based design for the implementation of a phase I/II trial with delayed onset binary outcomes for both safety and activity, in comparison to the existing methods. This exploration is motivated by the Targeted Alpha Therapy (TAT) platform. TAT is an emerging modality within the field of radiotherapy, combining tumor-targeting molecules linked to alpha particle-emitting radioisotopes that aim to offer a new approach to treating cancers and potentially overcoming resistance.

Several first in human studies have been initiated in recent years to support the development of TAT treatments targeting a broad range of cancer indications (U.S. National Library of Medicine 2015, 2018a, 2018b, 2020). Clinical and practical experience accumulated during the conduct of these trials has highlighted the importance of long-term toxicity and activity outcomes and therefore a potential benefit of incorporating these features into the statistical design.

In addition, the need for reliable designs for this purpose directly relates to one of the goals of the FDA’s Project Optimus (U.S. Food & Drug Administration 2023), to “develop strategies for dose finding and dose optimization that leverages nonclinical and clinical data in dose selection, including randomized evaluations of a range of doses in trials. An emphasis of such strategies will be placed on performing these studies as early as possible in the development program and as efficiently as possible to bring promising new therapies to patients.”

The article is outlined as follows. In Section 2 we introduce the methods and setting, presenting results of their application in Section 3. We consider a realistic setting with stopping and enforcement rules, a theoretical setting where these rules are relaxed, and a setting with varying activity time trends, before concluding with a discussion in Section 4.

## 2 Methods

In this section, we outline the proposed Joint TITE-CRM design, the model-assisted design of Liu and Johnson (2016), the *A_T_*/*A_E_* design of Yuan and Yin (2009) and the TITE-B design of Yan et al. (2019). We first consider the general setting of the trial, before detailing how the designs differ.

We consider the setting where there are *J* dose levels, $d_{1},\ldots ,d_{j},\ldots ,d_{J}$ available for exploration. Patients enter the trial in cohorts, with all patients in each cohort assigned to the same dose and followed up for *τ* cycles of treatment. A DLT (yes/no) may occur at any time during the follow up period, in which case the patient leaves the trial. The patient may also observe an activity response (yes/no) at any time during their follow up, which is censored at the time of DLT if the patient observes a DLT and no activity response. This censoring is used for all methods, since it is based on the assumption that the patient leaves the trial following a DLT response

The choice of dose to assign to the next cohort and the final dose recommendation are both chosen based on the design. For comparability, in each case, the trial proceeds in the following way:

1. The first cohort of patients is assigned to the lowest dose.
2. After one cycle of follow up, if no DLT is observed, escalate to next highest dose and continue after each cycle until a DLT is observed or the highest dose is reached. If a DLT is observed, the models from the design take over the dosing decision (there is no requirement on activity responses to start using the design model).
3. The relevant models are fitted to the currently observed responses (which will be from one cycle of follow up for the last cohort, two cycles for the last but one cohort etc.) and posterior distributions are updated.
4. A set of “admissible” doses are calculated based on the models fitted.
5. The best dose of the “admissible” set according to some criterion of the design is chosen to assign to the next cohort (subject to certain pre-specified rules discussed in Section 2.5).
6. After the next cycle of follow up, return to step 3. The trial is stopped when one of the pre-specified stopping rules is triggered.

It is worthwhile to note that the form of the prior and posterior in step 3, the admissible doses in step 4 and criterion in step 5 are the only aspects unique to each design.

### 2.1 Joint TITE-CRM

Based on the TITE-CRM (Cheung and Chappell 2000) and similar to the bCRM (Braun 2002), we modify the procedure to include both safety and activity, whilst keeping the structure of the TITE-CRM.

We use a two-parameter logistic model for both safety and activity outcomes:

$$\begin{array}{c} F(d,\beta_{A})=\frac{\;\text{exp}\;(\beta_{A,0}+\beta_{A,1}d)}{1+\;\text{exp}\;(\beta_{A,0}+\beta_{A,1}d)},\\ F(d,\beta_{T})=\frac{\;\text{exp}\;(\beta_{T,0}+\beta_{T,1}d)}{1+\;\text{exp}\;(\beta_{T,0}+\beta_{T,1}d)}, \end{array}$$

where $\beta_{A}=(\beta_{A,0},\beta_{A,1})$ and $\beta_{T}=(\beta_{T,0},\beta_{T,1})$ are the parameter vectors for activity and toxicity, respectively.

We then must give weights to both toxicity and activity observations based on their follow up times, and a weighted dose response model, *G*, is used for each:

$$\begin{array}{c} G(d,w^{\left(A\right)},\beta_{A})=w^{\left(A\right)}F(d,\beta_{A}),\\ G(d,w^{\left(T\right)},\beta_{T})=w^{\left(T\right)}F(d,\beta_{T}), \end{array}$$

where $0\leq w^{\left(A\right)},w^{\left(T\right)}\leq 1$ are functions of time-to-event of a patient response. We use the proposed weights of Cheung and Chappell (2000), that is the proportion of total follow-up. In this context, this simplifies to using $w_{i}^{\left(T\right)}=u_{i}/\tau$, where *u_i_* is the current number of cycles patient *i* has been observed for, unless a DLT is observed in which case $w_{i}^{\left(T\right)}=1$. In a similar fashion, we use $w_{i}^{\left(A\right)}=u_{i}/\tau$, where *u_i_* is the current number of cycles patient *i* has been observed for, unless an activity outcome is observed, in which case $w_{i}^{\left(A\right)}=1$. The exception to this is if a DLT is observed before an activity outcome can be observed, in which case the activity observation is censored by DLT time and so $w_{i}^{\left(A\right)}=(\text{DLT time ‐ entry time})/\tau$. We note that although here we use discrete numbers of cycles for the weights unless censoring occurs, this is a consequence of the setup of the trial and not the Joint TITE-CRM itself, which can use any weights.

We consider all binary combinations of toxicity and activity outcomes (as opposed to trinary sometimes used in such applications), related by a Gumbel Model (Thall and Cook 2004):

$$\begin{array}{c} \pi_{a,b}(d,\beta ,w)={\left(G(d,w^{\left(A\right)},\beta_{A})\right)}^{a}{\left(1-G(d,w^{\left(A\right)},\beta_{A})\right)}^{1-a}\\ {\left(G(d,w^{\left(T\right)},\beta_{T})\right)}^{b}{\left(1-G(d,w^{\left(T\right)},\beta_{T})\right)}^{1-b}+\\ {\left(-1\right)}^{a+b}(G(d,w^{\left(A\right)},\beta_{A}))(1-G(d,w^{\left(A\right)},\beta_{A}))\\ G(d,w^{\left(T\right)},\beta_{T})(1-G(d,w^{\left(T\right)},\beta_{T}))\left(\frac{e^{\psi }-1}{e^{\psi }+1}\right), \end{array}$$

where $\pi_{a,b}$ is the probability of observing each of the four combinations of binary activity outcome (*a* = 0 for no activity observed, *a* = 1 for activity observed) and binary toxicity outcome (*b* = 0 for no DLT observed, *b* = 1 for DLT observed), $\beta =(\beta_{A},\beta_{T}),\;w=(w^{\left(A\right)},w^{\left(T\right)})$ and ψ∈(−∞,∞) is a correlation parameter of the distribution, with positive values of *ψ* corresponding to a positive correlation between activity and toxicity, negative values corresponding to a negative correlation, and larger absolute values of *ψ* corresponding to stronger correlations.

The likelihood is then:

(1) $$L(\beta )=\prod_{i=1}^{n}\prod_{a=0}^{1}\prod_{b=0}^{1}\{\pi_{a,b}(d_{\left[i\right]},\beta ,w_{i}){\}}^{I(Y_{i}=(a,b))},$$

for patients i=1,…,n, where $d_{\left[i\right]}$ is the dose assigned to patient *i* and *Y_i_* is the observed response for patient *i*.

Priors are elicited on *ψ*, $\beta_{A,0},\;\beta_{A,1},\;\beta_{T,0},\;\beta_{T,1}$, and the likelihood (1) is used to update the joint posterior for all of the parameters using MCMC methods. The next dose is then chosen based on a utility function of *π_A_* and *π_T_*, the probability of activity and toxicity, respectively.

We use the following utility function, as a result of a sensitivity analysis conducted on this choice:

(2) $$U(\pi_{A},\pi_{T})=\pi_{A}-\omega_{1}\pi_{T}-\omega_{2}\pi_{T}\;I(\pi_{T}>\phi_{T}).$$

This uses two weights, *ω*_1_ and *ω*_2_ and a toxicity threshold $\phi_{T}$, whereby we penalize doses that have a higher probability of DLT than this threshold. The next dose is chosen to maximize the utility out of a set of admissible doses.

We varied the weights *ω*_1_ and *ω*_2_ in the linear utility function, and also consider the more complex utility contour method suggested by Thall and Cook (2004). It was found that there was negligible difference in the outcomes for any of the alternative utilities. This led to use using the simple linear utility used by Liu and Johnson (2016) in our implementation of the Joint TITE-CRM, with the same weights, *ω*_1_=0.33 and *ω*_2_=1.09.

This utility is simple to implement and to interpret. Using the same utility function for the two different designs also allows the results to be interpreted in terms of the inference used.

### 2.2 Model-Assisted Approach (Liu and Johnson 2016)

The model-assisted method by Liu and Johnson (2016) uses a Bayesian dynamic model for binary responses, *y*, as follows:

$$\begin{array}{c} y_{i}^{\left(T\right)}|d=d_{j}∼\text{Bern}(p_{T,j})\\ p_{T,j}=p_{T,j-1}+(1-p_{T,j-1})\beta_{T,j},j=2,\ldots J\\ p_{T,1}=\beta_{T,1}\\ \beta_{T,j}∼\text{Beta}(a_{T,j},b_{T,j})j=1,\ldots J. \end{array}$$

The subscript *T* indicates toxicity, with the equivalent for activity labeled *A*. This is described as a model-assisted approach since there is no dose–response model, and as such, the specification of the $p_{T,j}$ ensures monotonicity.

The likelihood is given as

$$\begin{array}{c} L(y|\beta_{T},\beta_{A})=\prod_{k=\{A,T\}}\prod_{i=1}^{n}{\left\{w_{i}^{\left(k\right)}\left(1-\prod_{r=1}^{j[i]}\left(1-\beta_{k,r}\right)\right)\right\}}^{y_{i}^{\left(k\right)}}\\ \;{\left\{1-w_{i}^{\left(k\right)}\left(1-\prod_{r=1}^{j[i]}\left(1-\beta_{k,r}\right)\right)\right\}}^{1-y_{i}^{\left(k\right)}}, \end{array}$$

where j[i] is the dose level assigned to patient *i*. This likelihood uses the same weights as in the Joint TITE-CRM and is used to update the posterior distribution. The same utility function as the Joint TITE-CRM (2) is used to assign the next dose.

### 2.3 *A_T_*/*A_E_* Design (Yuan and Yin 2009)

We here give an overview of the *A_T_*/*A_E_* design, for further details we refer the reader to the original proposal by Yuan and Yin (2009).

This design fits survival models to the time-to-event data for both toxicity (*t_T_*) and activity (*t_A_*) outcomes, assuming a Weibull distribution:

$$\begin{array}{c} S_{T}(t_{T}|d)=\;\text{exp}\;\{-\lambda_{T}t_{T}^{\alpha_{T}}\;\text{exp}\;(\beta_{T}d)\},\\ S_{A}(t_{A}|d)=\;\text{exp}\;\{-\lambda_{A}t_{A}^{\alpha_{A}}\;\text{exp}\;(\beta_{A}d)\},\\ S_{A}^{*}(t_{A}|d)=1-\pi +\pi S_{A}(t_{A}|d), \end{array}$$

where $S^{*}$ is the improper survival with the proportion of the population susceptible to the activity outcome denoted as *π*. This improper survival is therefore used to calculate the probability of observing activity outcomes at any given dose, as well as in the *A_T_*/*A_E_* criterion.

The bivariate time-to-event data is then modeled as

$$S(t_{T},t_{A}|d)={\left\{S_{T}{\left(t_{T}|d\right)}^{-1/\phi }+S_{A}{\left(t_{A}|d\right)}^{-1/\phi }-1\right\}}^{-\phi },$$

where ϕ is the correlation between the times to toxicity and activity. In order to compute the likelihood, define $y_{T}=\text{min}(t_{T},c_{T})$ and $\Delta_{T}=I(t_{T}\leq c_{T})$ where *c_T_* is the censoring time, and define *y_A_* and Δ*_A_* similarly for activity. The likelihood is then

$$L(\theta |\text{dat}a_{i})=L_{1}^{\Delta_{T}\Delta_{A}}L_{2}^{\Delta_{T}(1-\Delta_{A})}L_{3}^{(1-\Delta_{T})\Delta_{A}}L_{4}^{\left(1-\Delta_{T})(1-\Delta_{A}\right)},$$

where, for the *π* and *S* defined as above:

$$\begin{array}{c} L_{1}=\pi \frac{\partial^{2}S(y_{T},y_{A}|d)}{\partial y_{T}\partial y_{A}},\\ L_{2}=-(1-\pi )\frac{\partial S_{T}(y_{T}|d)}{\partial y_{T}}-\pi \frac{\partial S(y_{T},y_{A}|d)}{\partial y_{T}},\\ L_{3}=-\pi \frac{\partial S(y_{T},y_{A}|d)}{\partial y_{A}},\\ L_{4}=(1-\pi )S_{T}(y_{T}|d)+\pi S(y_{T},y_{A}|d). \end{array}$$

The parameters *λ_A_*, *α_A_*, *β_A_*, *λ_T_*, *α_T_*, and *β_T_* are assigned independent gamma priors and *π* and ϕ are assigned uniform priors. The joint posterior distribution is then updated using a Gibbs Sampler.

The criterion used for decision making is a ratio of the area under the curve (AUC) for the survival for toxicity and activity:

$$\frac{A_{T}}{A_{E}}=\frac{\alpha_{T}^{-1}{\left\{\lambda_{T}\;\text{exp}\;(\beta_{T}d)\right\}}^{-1/\alpha_{T}}\Gamma \{\alpha_{T}^{-1},\lambda_{T}\;\text{exp}\;(\beta_{T}d)\tau^{\alpha_{T}}\}}{\begin{array}{c} (1-\pi )\tau +\pi \alpha_{A}^{-1}{\left\{\lambda_{A}\;\text{exp}\;(\beta_{A}d)\right\}}^{-1/\alpha_{A}}\\ \Gamma \{\alpha_{A}^{-1},\lambda_{A}\;\text{exp}\;(\beta_{A}d)\tau^{\alpha_{A}}\} \end{array}},$$

where Γ(a,b) is the incomplete gamma function and *τ* is the follow up time. The dose in the admissible dose set that maximizes the *A_T_*/*A_E_* is chosen for the next cohort of patients.

### 2.4 TITE-B Design (Yan et al. 2019)

The TITE-B design by Yan et al. (2019) also expands the TITE-CRM to include late-onset activity endpoints, however, the approach of both estimating the probabilities of toxicity and activity and choosing the OBD is different to that of the proposed Joint TITE-CRM. The TITE-B uses a one-parameter power model for both toxicity and activity, and fits the models independently of one another. The toxicity model is

$$\psi (d_{j},\theta )=p_{j}^{\;\text{exp}\;(\theta )},$$

where $p_{1}<p_{2}<\cdots p_{J}<1$ are pre-specified constants of the skeleton, corresponding to doses $d_{1},\ldots ,d_{J}$. Using the same weights as in the Joint TITE-CRM, the following likelihood is constructed:

$$L(\theta )=\prod_{i=1}^{n}w_{i}^{\left(T\right)}\psi {\left(d_{\left[i\right]},\theta \right)}^{y_{i}^{\left(T\right)}}{\left(1-w_{i}^{\left(T\right)}\psi (d_{\left[i\right]},\theta )\right)}^{1-y_{i}^{\left(T\right)}}.$$

The posterior distribution for *θ* is updated using this likelihood and the prior distribution for *θ*, and used to calculate the posterior estimate of toxicity at each dose. For activity, multiple working models, labeled l=1,…,L, are used:

$$\phi_{l}(d_{j},\beta_{l})=q_{jl}^{\;\text{exp}\;(\beta_{l})},$$

with $q_{jl}$ being the pre-specified constants in the skeleton for working model l. Again, using the same weights as in the Joint TITE-CRM, the following likelihood is constructed for each working model l=1,…,L:

(3) $$L(\beta_{l})=\prod_{i=1}^{n}w_{i}^{\left(A\right)}\phi {\left(d_{\left[i\right]},\beta_{l}\right)}^{y_{i}^{\left(A\right)}}{\left(1-w_{i}^{\left(A\right)}\phi (d_{\left[i\right]},\beta_{l})\right)}^{1-y_{i}^{\left(A\right)}}.$$

Prior probabilities h(1),…,h(L) are assigned to each of the working models, and the posterior probability of each working model is calculated:

$$\kappa_{l}=\frac{h(l)\int L(\beta_{l})g(\beta_{l})d\beta_{l}}{\sum_{l=1}^{L}h(l)\int L(\beta_{l})g(\beta_{l})d\beta_{l}},$$

where $g(\beta_{l})$ is the prior distribution elicited on $\beta_{l}$.

The criterion used to make the next dose recommendation is to first define a set of safe doses using the toxicity model, such that the posterior estimate of toxicity is below a pre-specified level, then to randomize the dosing choice from this set based on the posterior probabilities of a subset of the working models and the recommended dose from each candidate model, S(l). The randomization probability, $R_{j}^{*}$, for dose *d_j_* is given as

$$\begin{array}{c} R_{j}^{*}=\frac{R_{j}^{**}}{\sum_{j=1}^{J}R_{j}^{**}}\text{where}\\ R_{j}^{**}=\sum_{l=1}^{2J-1}\kappa_{n}(l)\;I(d_{j}=S(l)\text{and}\kappa_{n}(l)\geq \kappa_{\left(L-L^{\prime }+1\right)}). \end{array}$$

Here, $\kappa_{\left(1\right)}\leq \cdots \leq \kappa_{\left(L\right)}$ denote the ordered posterior model probabilities $\kappa_{n}(l)$. L=2J−1 is the total number of working models and $L^{\prime }$ is the number of candidate models considered. The number of candidate models used in the randomization reduces as more patients enter the trial:

$$L^{\prime }=\left\lceil {\left(\frac{N-n}{N}\right)}^{\delta }L\right\rceil ,$$

where *δ* is a pre-specified constant.

### 2.5 Rules

As well as ensuring any assigned dose is admissible, we also apply a set of enforcement and stopping rules. For any given dose *d_j_*, $p_{1,d_{j}}$ is the P(DLT) in the first cycle, $p_{\tau ,d_{j}}$ is the P(DLT) in the full follow up of *τ*.

#### 2.5.1 Admissible Dose Set

For the Joint TITE-CRM, Model-Assisted Approach and *A_T_*/*A_E_* design, the admissible dose set is calculated as all doses satisfying the following criteria:

$$P(\pi_{T}<\pi_{T}^{*})>q_{T}\text{ \& }P(\pi_{A}>\pi_{A}^{\text{*}})>q_{A}.$$

for some thresholds *q_T_* and *q_A_*, where $\pi_{T}^{*}$ and $\pi_{A}^{*}$ are the target probabilities for toxicity and activity in the entire follow-up *τ*. These constraints ensure that the escalation proceeds to a promising dose that is considered neither futile nor unsafe.

For the TITE-B design, following the specification by Yan et al. (2019), the admissible set is defined only by safety, and as those doses that satisfy ${\hat{\pi }}_{T}(d_{j})<\xi$, where ${\hat{\pi }}_{T}(d_{j})$ is the estimate of DLT rate at dose *d_j_* and *ξ* is the maximum acceptable DLT rate for the entire follow up.

#### 2.5.2 Enforcement Rules

Enforcement rules are to ensure the safety within the trial. The hard safety rule ensures safety in the first cycle of treatment, and as such, the safety cutoff of 0.3 is used as opposed to $\pi_{T}^{*}$. The K-fold skipping doses rule ensures escalation is not too aggressive and is based on dose values as opposed to levels so that it is meaningful for the model-based and model-assisted designs.

1. **Hard Safety**: This rule ensures that if there is a very high probability that the toxicity of an experimented dose exceeds 0.3 in the first cycle, then that dose and all above are excluded from any further experimentation (i.e., dose *d_j_* and all above are excluded when $P(p_{1,d_{j}}>0.3)>\zeta$ for some threshold *ζ*). In this implementation we use a threshold for excessive toxicity of ζ=0.95, with a Beta(1,1) prior for Binomial responses. This is equivalent to the case where there are at least 3 DLT responses out of 3 patients, at least 4 DLT responses out of 6 patients, or at least 5 DLT responses out of 9 patients, then all dose assignments must be strictly lower than that dose for the rest of the study. If the lowest dose is excluded then the trial stops with no dose recommendation made.
2. **K-fold Skipping Doses**: The next dose assignment must be no more than a 2-fold increase in the value of the highest experimented dose so far.

#### 2.5.3 Stopping Rules

Stopping rules are to define when we may stop the trial. This may be because we have a level of certainty about the estimated OBD or for futility/safety issues. Either that the dose range is unsafe and hence entirely too high, the dose range is all below target toxicity and hence entirely too low, or that the dose range is all below target activity. It is useful to be able to recommend stopping the trial when all doses are deemed too safe so that a new dose set can be established and investigated.

1. **No Admissible Doses**: If no doses satisfy the two constraints in Section 2.5.1, then the trial is stopped, either for futility, safety or both.
2. **Lowest Dose Deemed Unsafe**: If $P(p_{1,d_{1}}>30\%)>0.80$ and at least one cohort of patients has been assigned to dose *d*_1_, the trial is stopped.
3. **Highest Dose Deemed Very Safe**: If $P(p_{1,d_{J}}\leq 30\%)>0.80$ and at least one cohort of patients has been assigned to dose *d_J_*, the trial is stopped.
4. **Sufficient Information**: For a pre-defined cutoff value, *C_suff_*, if a dose is recommended for the next cohort on which *C_suff_* patients have already been assigned in the escalation, the trial is stopped.
5. **Precision**: If the safety and activity profile are both estimated precisely enough, the trial is stopped. This precision is defined as CV(MTD)<30% and $\text{CV}(d_{\left[\pi_{A}^{*}\right]})<30\%$, with the coefficient of variation calculated as an adjusted median absolute deviation divided by the median and $d_{\left[\pi_{A}^{*}\right]}$ is the dose associated with the target activity. Both of these must be satisfied before the stopping rule is enforced. This stopping rule is only used once at least *C_suff_* patients have had at least one cycle of treatment in the escalation, on any dose. This rule is not applicable for the model-assisted method.
6. **Hard Safety**: If the lowest dose is considered unsafe according to the hard safety enforcement rule, the trial is stopped.
7. **Maximum Patients**: If the maximum number of patients ($n=n_{\text{max}}$) have been recruited, the trial is stopped.

We consider two settings; a realistic setting where all enforcement and stopping rules are applied, and a theoretical setting where stopping rules 2, 3, 4, 5 are not applied. This second setting is to investigate the behavior of the designs without restrictions.

## 3 Simulations

In order to compare the Joint TITE-CRM to the model-assisted design, the *A_T_*/*A_E_* design and the TITE-B design, we conduct simulation studies in a range of scenarios. For comparison, we also consider a non-time-to-event method, the Joint CRM. For this implementation, all *τ* cycles must be observed from the previous cohort before the next cohort is assigned. This is equivalent to setting $w^{\left(A\right)}=w^{\left(T\right)}=1$ for each patient in the Joint TITE-CRM, effectively removing the time-to-event element of the design.

### 3.1 Setting

We consider the setting where six doses are investigated: 1.5MBq, 2.5MBq, 3.5MBq, 4.5MBq, 6.0MBq, 7.0MBq with a follow-up period of *τ* = 3 cycles, with each cycle lasting 6 weeks. The trial proceeds as outlined in Section 2, with cohorts of size 3. In addition the following parameter values are used for the rules: $n_{\text{max}}=60$ and *C_suff_* = 30.

We specify $\pi_{T}^{*}=0.391$ as the target probability of DLT for the full follow up period of 3 cycles, corresponding to P(DLT)=0.3 in cycle 1. Following Barnett et al. (2022), this assumes the probability of DLT decreases by a factor of 1/3 in subsequent cycles, conditional on no DLT in the previous cycles. Note that this is also reflective of cumulative toxicity, in line with results from Altzerinakou, Collette, and Paoletti (2019). This choice is made to represent a realistic setting, and can be different based on assumptions on the setting.

We use a combination of five safety scenarios (T1, T2, T3, T4, T5) and four activity scenarios (A1, A2, A3, A4), giving 20 scenarios in total. We then extend to consider different activity patterns in Section 3.6. The five safety scenarios represent four scenarios where there is at least one safe dose and one where no doses are safe. In T1, all doses are safe; in T2, only the highest dose is unsafe; in T3, dose levels 4 and above are unsafe; in T4, dose levels 2 and above are unsafe; and in T5 all doses are unsafe. The four activity scenarios are described by the probability of activity in the whole follow up period, with the lower bound on target activity as $\pi_{A}^{*}=0.2$. In A1, all dose levels are active with a plateau reached at dose level 3; in A2, all dose levels are active, starting at a lower activity at dose 1 than A1 and increasing with dose; in A3, dose levels 3 and above are active; and in A4, no dose levels are active. Scenarios with lower numbers are therefore most safe/active. Here we assume the probability of activity in the total follow up is three times the probability of activity in the first cycle. In Section 3.6, we alter this assumption.

Scenarios are referred to as Tx.Ay where x is the safety scenario, y is the activity scenario. Table 1 gives the individual safety and activity scenarios, while Table 2 gives the utility and *A_T_*/*A_E_* values for each of the 20 combination scenarios.

**Table 1:** Scenario definitions for activity and toxicity.

Safety	1.5MBq	2.5MBq	3.5MBq	4.5MBq	6.0MBq	7.0MBq
T1 (cycle 1)	0.100	0.120	0.140	0.160	0.180	0.200
T1 (full follow up)	0.140	0.166	0.193	0.219	0.245	0.270
T2 (cycle 1)	0.100	0.130	0.160	0.200	0.250	0.400
T2 (full follow up)	0.140	0.180	0.219	0.270	0.332	0.503
T3 (cycle 1)	0.100	0.200	0.300	0.400	0.500	0.600
T3 (full follow up)	0.140	0.270	0.391	0.503	0.606	0.701
T4 (cycle 1)	0.300	0.400	0.450	0.500	0.550	0.600
T4 (full follow up)	0.391	0.503	0.556	0.606	0.655	0.701
T5 (cycle 1)	0.400	0.450	0.500	0.550	0.600	0.650
T5 (full follow up)	0.503	0.556	0.606	0.655	0.701	0.746
Activity	1.5MBq	2.5MBq	3.5MBq	4.5MBq	6.0MBq	7.0MBq
A1	0.300	0.400	0.500	0.500	0.500	0.500
A2	0.200	0.300	0.400	0.500	0.600	0.700
A3	0.100	0.150	0.200	0.300	0.500	0.700
A4	0.100	0.120	0.140	0.160	0.180	0.200

**Table 2:** Utility for the 20 scenarios, with OBD highlighted in bold and acceptable doses highlighted in *italics.*

Scenario	1.5MBq	2.5MBq	3.5MBq	4.5MBq	6.0MBq	7.0MBq
T1.A1 Utility	*0.25*	*0.35*	*0.44*	*0.43*	*0.42*	*0.41*
T1.A1 *A_T_*/*A_E_*	*1.05*	*1.10*	*1.14*	*1.12*	*1.09*	*1.06*
T2.A1 Utility	*0.25*	*0.34*	**0.43**	0.41	0.39	–0.22
T2.A1 *A_T_*/*A_E_*	*1.05*	*1.08*	**1.12**	1.06	0.99	0.79
T3.A1 Utility	*0.25*	*0.31*	**0.37**	–0.22	–0.36	–0.50
T3.A1 *A_T_*/*A_E_*	*1.05*	*0.99*	**0.92**	0.79	0.66	0.53
T4.A1 Utility	**0.17**	–0.32	–0.29	–0.36	–0.43	–0.50
T4.A1 *A_T_*/*A_E_*	**0.81**	0.74	0.72	0.66	0.59	0.53
T5.A1 Utility	–0.42	–0.39	–0.36	–0.43	–0.50	–0.56
T5.A1 *A_T_*/*A_E_*	0.69	0.67	0.66	0.59	0.53	0.46
T1.A2 Utility	*0.15*	*0.25*	*0.34*	*0.43*	*0.52*	*0.61*
T1.A2 *A_T_*/*A_E_*	*0.99*	*1.03*	*1.07*	*1.12*	*1.17*	*1.23*
T2.A2 Utility	*0.15*	*0.24*	*0.33*	*0.41*	**0.49**	–0.02
T2.A2 *A_T_*/*A_E_*	*0.99*	*1.02*	*1.05*	*1.06*	**1.07**	0.92
T3.A2 Utility	*0.15*	*0.21*	**0.27**	–0.22	–0.26	–0.30
T3.A 2*A_T_*/*A_E_*	*0.99*	*0.93*	**0.86**	0.79	0.71	0.61
T4.A2 Utility	*0.07*	–0.42	–0.39	–0.36	–0.33	–0.30
T4.A2 *A_T_*/*A_E_*	*0.76*	0.69	0.67	0.66	0.64	0.61
T5.A2 Utility	–0.52	–0.49	–0.46	–0.43	–0.40	–0.36
T5.A2 *A_T_*/*A_E_*	0.65	0.63	0.61	0.59	0.57	0.54
T1.A3 Utility	0.05	0.10	*0.14*	*0.23*	*0.42*	*0.61*
T1.A3 *A_T_*/*A_E_*	0.94	0.94	*0.95*	*0.98*	*1.09*	*1.23*
T2.A3 Utility	0.05	0.09	*0.13*	*0.21*	**0.39**	–0.02
T2.A3 *A_T_*/*A_E_*	0.94	0.93	*0.92*	*0.93*	**0.99**	0.92
T3.A3 Utility	0.05	0.06	*0.07*	–0.42	–0.36	–0.30
T3.A3 *A_T_*/*A_E_*	0.94	0.85	*0.76*	0.69	0.66	0.61
T4.A3 Utility	–0.03	–0.57	–0.59	–0.56	–0.43	–0.30
T4.A3 *A_T_*/*A_E_*	0.72	0.64	0.60	0.58	0.59	0.61
T5.A3 Utility	–0.62	–0.64	–0.66	–0.63	–0.50	–0.36
T5.A3 *A_T_*/*A_E_*	0.62	0.58	0.54	0.52	0.53	0.54
T1.A4 Utility	0.05	0.07	0.08	0.09	0.10	*0.11*
T1.A4 *A_T_*/*A_E_*	0.94	0.93	0.92	0.90	0.89	*0.88*
T2.A4 Utility	0.05	0.06	0.07	0.07	0.07	–0.52
T2.A4 *A_T_*/*A_E_*	0.94	0.92	0.89	0.86	0.81	0.65
T3.A4 Utility	0.05	0.03	0.01	–0.56	–0.68	–0.80
T3.A4 *A_T_*/*A_E_*	0.94	0.84	0.74	0.64	0.54	0.44
T4.A4 Utility	–0.03	–0.60	–0.65	–0.70	–0.75	–0.80
T4.A4 *A_T_*/*A_E_*	0.72	0.63	0.58	0.53	0.48	0.44
T5.A4 Utility	–0.62	–0.67	–0.72	–0.77	–0.82	–0.86
T5.A4 *A_T_*/*A_E_*	0.62	0.57	0.53	0.48	0.43	0.38

NOTE: The *A_T_*/*A_E_* criteria has no penalty for exceeding a safety threshold and hence may give higher values for unsafe doses.

In Table 2, it can be seen that the utility criterion and *A_T_*/*A_E_* criterion do not always agree on the OBD. For example in scenarios T3.A2 and T3.A1, the 3.5MBq dose is the OBD according to the utility criterion, and the 1.5MBq dose is the OBD according to the *A_T_*/*A_E_* criterion. We have chosen to designate the 3.5MBq dose as the true OBD as this is more realistic, with a P(DLT) equal to target and a higher P(Activity) than the lower dose. This does, however, highlight an issue concerning the *A_T_*/*A_E_* criteria, in that in maximizing the ratio between the two areas under the curves, it does not target any specific safety level. Hence, when ratios are similar across doses, even when values are not, there may be difficulties in selection of the optimal dose.

### 3.2 Data Generation

To generate the event times for toxicities and activity, *t_T_* and *t_A_*, we use a model that is not based on the assumptions of any of the methods. We use a bivariate log-normal *Lognormal*_2_) distribution for event times, with parameters matched to the first cycle and full follow up probabilities of toxicity and activity, with a correlation parameter of–1/2. In the range of scenarios considered, all implicitly assume a positive association between activity and toxicity. The negative association is between activity and toxicity event times only, under the specifications of the probabilities described. This reflects the likelihood that early activity outcomes are associated with DLT outcomes later, although this is not a strong association. This is a simple yet effective mechanism to generate data that allows for easy specification of probabilities at dose levels that do not themselves follow any specific parametric dose-response model.

$$\left(\begin{array}{c} t_{T}\\ t_{A} \end{array}\right)∼{\text{Lognormal}}_{2}\left(\left(\begin{array}{c} \mu_{T}\\ \mu_{A} \end{array}\right),\left(\begin{array}{cc} \sigma_{T}^{2} & -\frac{\sigma_{T}\sigma_{A}}{2}\\ -\frac{\sigma_{T}\sigma_{A}}{2} & \sigma_{A}^{2} \end{array}\right)\right).$$

### 3.3 Prior Distributions

Since all methods are Bayesian, we must specify prior distributions for the parameters of the models.

#### 3.3.1 Joint TITE-CRM and Joint CRM

Here we use priors

$$\left(\begin{array}{c} \beta_{0}\\ \;\text{log}\;(\beta_{1}) \end{array}\right)∼N_{2}\left(\left(\begin{array}{c} c_{1}\\ c_{2} \end{array}\right),\left(\begin{array}{cc} v_{1} & 0\\ 0 & v_{2} \end{array}\right)\right).$$

for both toxicity and activity. Values of hyper-parameters used are: $c_{1,T}=\;\text{log}\;(1/16),\;c_{2,T}=\;\text{log}\;(1/4),\;v_{1,T}=1,\;v_{2,T}=2$ and $c_{1,A}=-3,\;c_{2,A}=-0.2,\;v_{1,A}=1,\;v_{2,A}=1$. The values for toxicity are calibrated over a range of scenarios (see Mozgunov et al. 2021). The values for activity are chosen so that all doses have prior mean $(\pi_{A})>$ 0.3 and this increases with dose, with average effective prior sample size of one patient per dose level. We used a vague prior for ψ∼N(0,100).

#### 3.3.2 Model-Assisted Approach (Liu and Johnson 2016)

Here we use the priors $a_{A}=(0.2,0.3,0.4,0.45,0.5,0.6),\;b_{A}=(0.95$, 0.9, 0.8, 0.75, 0.7, $0.65),\;a_{T}=(0.05$, 0.1, 0.2, 0.25, 0.3, 0.35) and $b_{T}=(0.95$, 0.9, 0.8, 0.75, 0.7, 0.65), as suggested by Liu and Johnson (2016).

#### 3.3.3 *A_T_*/*A_E_* Design (Yuan and Yin 2009)

Here, we follow the priors implemented by Yuan and Yin (2009), with some minor modification to fit our setting. *λ_A_*, *α_A_*, *β_A_*, *λ_T_*, *α_T_*, and *β_T_* are assigned independent Gamma priors with shape and rate parameters of 0.1. These are constrained to be less than 3, 4, 1, 2, 3, and 1, respectively. The modification is of the upper bound of the *β* to account for the differing values of doses in our implementation to the original implementation. The uniform priors for *π* and ϕ are π∼U(0.6,1) and ϕ∼U(0,5).

#### 3.3.4 TITE-B Design (Yan et al. 2019)

For both *θ* and $\beta_{l}$, a N(0,1.34) prior is used. Following Yan et al. (2019), the skeleton for the toxicity model is (0.02, 0.06, 0.12, 0.20, 0.30, 0.40) and the *L* = 11 efficacy skeletons used are:

$$Q=\left(\begin{array}{c} q_{1}\\ q_{2}\\ q_{3}\\ q_{4}\\ q_{5}\\ q_{6}\\ q_{7}\\ q_{8}\\ q_{9}\\ q_{\mathrm{10}}\\ q_{\mathrm{11}} \end{array}\right)=\left(\begin{array}{cccccc} 0.59 & 0.50 & 0.40 & 0.30 & 0.20 & 0.12\\ 0.50 & 0.59 & 0.50 & 0.40 & 0.30 & 0.20\\ 0.40 & 0.50 & 0.59 & 0.50 & 0.40 & 0.30\\ 0.30 & 0.40 & 0.50 & 0.59 & 0.50 & 0.40\\ 0.20 & 0.30 & 0.40 & 0.50 & 0.59 & 0.50\\ 0.12 & 0.20 & 0.30 & 0.40 & 0.50 & 0.59\\ 0.20 & 0.30 & 0.40 & 0.50 & 0.59 & 0.59\\ 0.30 & 0.40 & 0.50 & 0.59 & 0.59 & 0.59\\ 0.40 & 0.50 & 0.59 & 0.59 & 0.59 & 0.59\\ 0.50 & 0.59 & 0.59 & 0.59 & 0.59 & 0.59\\ 0.59 & 0.59 & 0.59 & 0.59 & 0.59 & 0.59 \end{array}\right)$$

Equal prior weights are given to all activity working models, and a value of *δ* = 2 is used to determine the number of candidate models to include in the randomization.

### 3.4 Results

**Fig. 1:**
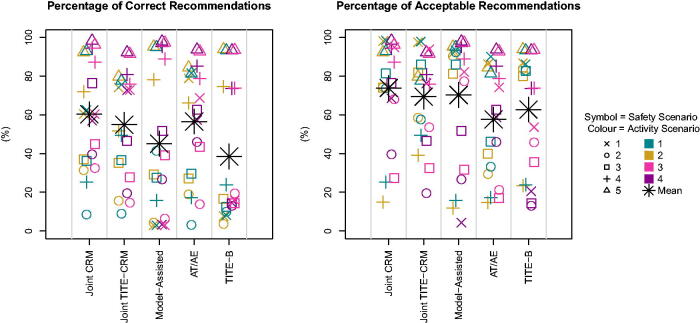
Proportion of correct and acceptable selections across scenarios.

We conducted 1000 simulations in each of the 20 scenarios. Figure 1 shows the percentage of simulations that select the true OBD if there is one, or the trial is stopped correctly when no true OBD exists in the dose set. The most noticeable feature is that the model-assisted method and the TITE-B design give a lower proportion of the correct recommendations than the two other methods that account for late-onset outcomes (Joint TITE-CRM and *A_T_*/*A_E_*). In scenarios where all doses are very safe, the model-assisted method performs poorly, a reflection on the cautious escalation often apparent in model-assisted designs. The TITE-B design also finds such scenarios challenging. The overall performance of the other two model based methods is similar, although with some differences in individual scenarios. For example the performance in T4.A1, where the lowest dose is the OBD, the Joint TITE-CRM method vastly outperforms the *A_T_*/*A_E_* design, which stops too often early for no admissible doses.

Interestingly, the percentage of acceptable selections does not follow the same pattern (Figure 1). Here we define an “acceptable” selection as one that is both safe and active, regardless of if it is the best in terms of the utility criterion. The Joint TITE-CRM is the best performing overall out of the designs accounting for late-onset outcomes. The model-assisted method performs poorly for example in T1.A4, where the acceptable selections are either the highest dose or stopping because the highest dose is too safe.

**Fig. 2:**
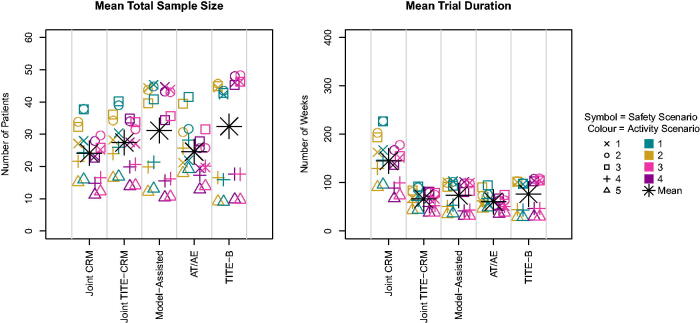
Mean sample size and trial duration across scenarios.

In terms of sample size, the model assisted method and TITE-B have a higher sample size on average across scenarios, as shown in Figure 2 while the other three methods have very similar sample sizes.

Although the performance of the Joint CRM is similar to that of the Joint TITE-CRM, with some slight advantages, this is at the cost of a much greater average trial length. Figure 2 indicates this relationship, with an average trial duration of more than twice that of the Joint TITE-CRM.

**Fig. 3:**
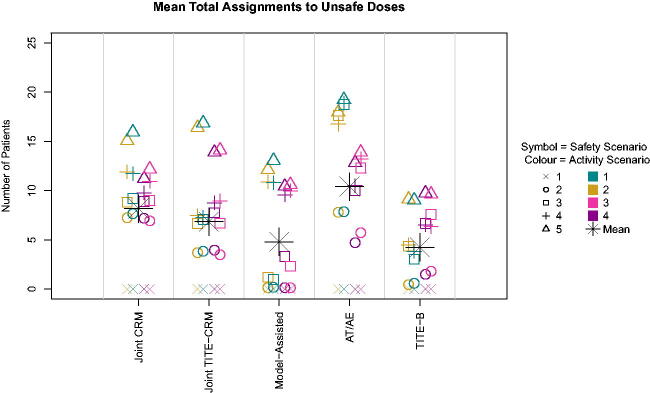
Number of patients assigned to unsafe doses across scenarios.

The patterns are similar between the model-based methods in terms of total sample sizes, correct and acceptable selections, however, there is a difference in the number of patients treated at unsafe doses, shown in Figure 3, with the *A_T_*/*A_E_* method assigning more patients to unsafe doses than both other methods in almost all scenarios. The model assisted method and TITE-B have much fewer patients assigned to unsafe doses, due to the cautious escalation behavior of model-assisted methods and the more strict safety criterion of the TITE-B.

The overall performance of the five methods in the setting of realistic stopping and enforcement rules can be summarized as follows. The cautious escalation of the model-assisted method gives rise to a poorer performance in terms of selections of correct and acceptable doses, with larger sample sizes and fewer patients exposed to unsafe doses, with the TITE-B having similar operating characteristics. The other two model based designs that account for late onset outcomes perform better, with similar percentages of correct and acceptable recommendations as each other, but with the *A_T_*/*A_E_* design assigning more patients to unsafe doses, showing a more aggressive escalation that is not rewarded with better selection performance. Additionally, the *A_T_*/*A_E_* design is substantially more complex and computationally intensive than the other methods, a cost that does not increase the performance. All four approaches considering partial information are vastly superior to the approach not allowing for such information in terms of trial duration.

### 3.5 Relaxed Stopping Rules

**Fig. 4:**
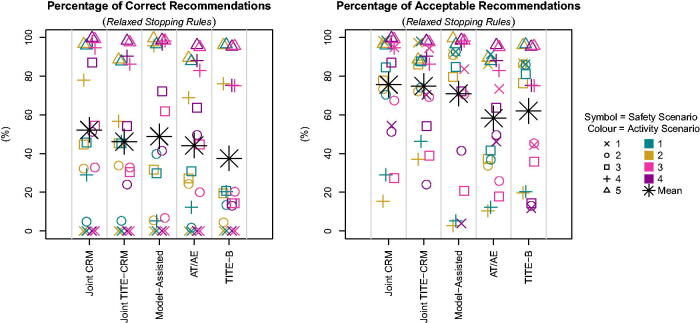
Proportion of correct and acceptable selections across scenarios with relaxed stopping rules.

When the stopping rules are relaxed to investigate the operating characteristics of the designs more closely, note that since stopping rule 3 is no longer enforced, there is no longer a correct selection in T1 and so all methods give 0% correct selection. We see in Figure 4 that in most scenarios where there is a true OBD (e.g., T2.A2, T4.A1, T2.A3) the Joint TITE-CRM gives a better performance of correct selections. When there are no admissible doses (e.g., T3.A4, T2.A4) the Joint TITE-CRM performs worse than the *A_T_*/*A_E_* design and the model assisted approach, but better than the TITE-B design.

It is noticeable that when considering acceptable selections, the Joint TITE-CRM performs the best out of the methods accounting for partial information in most scenarios. In the absence of the early stopping rules, this provides evidence that the Joint TITE-CRM is benefiting from the model-based inference that the model-assisted method lacks. However, the *A_T_*/*A_E_* method and TITE-B design also perform worse than the Joint TITE-CRM. For the *A_T_*/*A_E_* method, this may be due to the deviations from assumptions on the survival model used in the inference and data generation. For the TITE-B method, activity scenarios where many or all doses are not active give a low percentage of acceptable recommendations, since the design weights the dose recommendation based on activity, and does not specify an activity target or include activity in the choice of admissible doses.

**Fig. 5:**
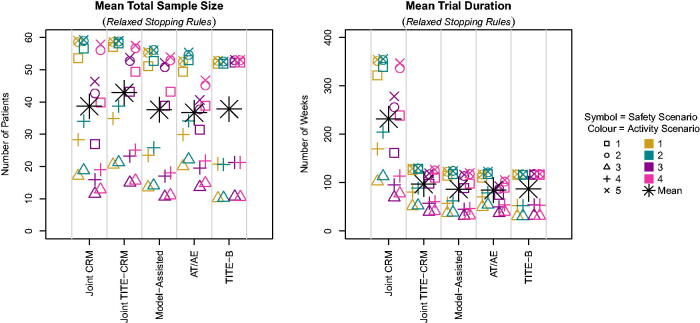
Mean sample size and trial duration across scenarios with relaxed stopping rules.

Figure 5 shows that the sample size is of course considerably larger when the stopping rules are relaxed, with many scenarios reaching nearly the maximum sample size on average. The Joint TITE-CRM has a larger sample size on average, which tallies up to the observation that this design had fewer correct stoppings for no admissible doses. This suggests that in general, this method is more willing to label a dose as admissible than the other methods.

The mean trial duration is also drastically increased for all methods, with the Joint CRM having an average duration of up to seven years, compared to the other methods giving less than three years.

**Fig. 6:**
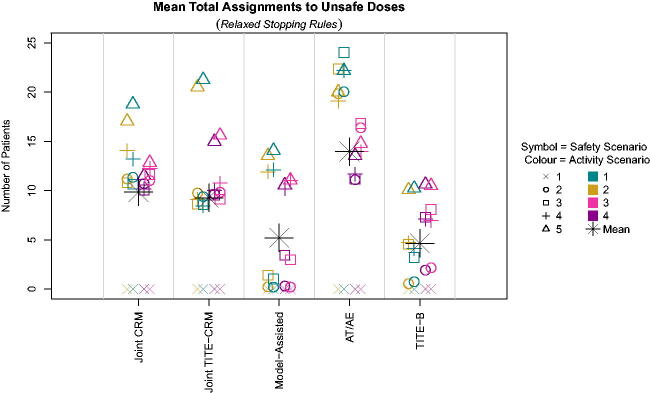
Number of patients assigned to unsafe doses across scenarios with relaxed stopping rules.

With the relaxed stopping rules, the *A_T_*/*A_E_* method once again has a large number of patients assigned to unsafe doses, owing to a more aggressive escalation, as seen in Figure 6. The model-assisted method and the TITE-B have much fewer patients assigned to unsafe doses, due to the more cautious escalation.

### 3.6 Activity Time Trend

In the main evaluation above, the data generation of the activity times assumed that the probability of activity in the first cycle is one third of the probability of activity in the entire follow up of three cycles. However, it may be the case that this is actually higher or lower than one third.

To investigate the effect of activity occurring at various points in the follow up, we consider three activity patterns. In activity pattern 1, the probability of activity in cycle 1 is 1/3 of the total probability; in activity pattern 2, it is 1/6 and in activity pattern 3 in is 1/2. This is to investigate the effect of varying time trends in activity.

It is possible that this change in activity time trend will affect the performance of the three methods that use the time-to-event outcomes, and so we investigate the difference that this can make in a selection of scenarios.

**Fig. 7:**
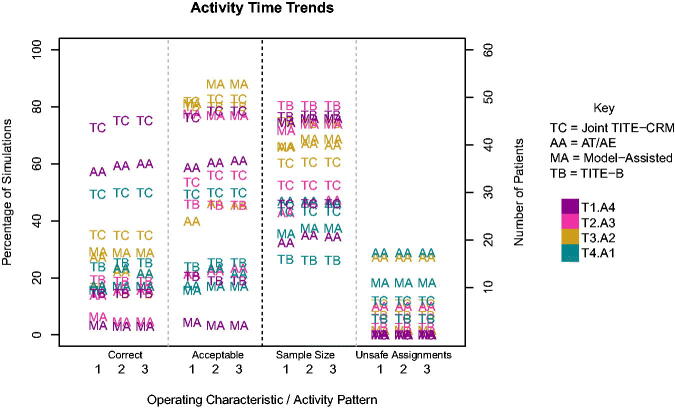
Operating characteristics for the three activity time trends.

Figure 7 shows the operating characteristics of the four time-to-event methods when the time trend is varied. The Joint CRM is not included here since it cannot account for time trends. There is no difference in the number of patients assigned to unsafe doses, and very little difference to the overall sample size. In terms of selections, the *A_T_*/*A_E_* design is most variable to the time trend, since this method is more heavily dependent on the survival curve. The Joint TITE-CRM, model-assisted method of Liu & Johnson and TITE-B are more robust to activity time trends.

## 4 Discussion

In this work, we have compared our designs for a dose-finding trial that uses both toxicity and activity outcomes in the form of time-to-event responses. The comparison has highlighted the impact of both the inference of dose–response relationship and the decision criteria. The challenge presented by a trial using both toxicity and activity outcomes is compounded by the late onset nature of the responses.

The decision criteria used in the *A_T_*/*A_E_* design, whilst intuitive for survival outcomes, does somewhat deviate from the objective of the dose-finding trial. Without penalty for unsafe doses, it gave an aggressive escalation path in many scenarios. This leads on from the point made regarding Table 2, that without targeting any safety level, scenarios with similar true ratios across levels present very challenging for this design. Additionally, the increased computational intensity of this design is not rewarded by an increase in performance.

The decision criteria used in the TITE-B design is less complex than the *A_T_*/*A_E_* design, but without specification of minimum efficacy, or requiring closeness to target toxicity, it is more challenging to recommend the true OBD. The stricter safety criteria for the admissible dose set leads to a more cautious escalation.

The model-assisted method and the Joint TITE-CRM implemented both used the same utility criterion, which allowed a comparison between the simple inference of Liu and Johnson (2016) and the joint logistic model. Here the increase in complexity is rewarded with increased performance. The Joint TITE-CRM is recommended as an alternative that balances complexity and performance across a range of scenarios.

## Data Availability

Software in the form of R code used to produce the presented results is available at https://github.com/helenyb/DF_Late_Activity_Safety
